# Negative Effect of Age, but Not of Latent Cytomegalovirus Infection on the Antibody Response to a Novel Influenza Vaccine Strain in Healthy Adults

**DOI:** 10.3389/fimmu.2018.00082

**Published:** 2018-01-29

**Authors:** Sara P. H. van den Berg, Albert Wong, Marion Hendriks, Ronald H. J. Jacobi, Debbie van Baarle, Josine van Beek

**Affiliations:** ^1^Centre for Infectious Disease Control, National Institute for Public Health and the Environment, Bilthoven, Netherlands; ^2^Laboratory of Translational Immunology, Department of Immunology, University Medical Center Utrecht, Utrecht University, Utrecht, Netherlands; ^3^Department of Statistics, Informatics and Mathematical Modelling, National Institute for Public Health and the Environment, Bilthoven, Netherlands

**Keywords:** cytomegalovirus, influenza vaccine, aging, immunosenescence, pandemic, antibody response, *de novo* immune response

## Abstract

Older adults are more vulnerable to influenza virus infection and at higher risk for severe complications and influenza-related death compared to younger adults. Unfortunately, influenza vaccine responses tend to be impaired in older adults due to aging of the immune system (immunosenescence). Latent infection with cytomegalovirus (CMV) is assumed to enhance age-associated deleterious changes of the immune system. Although lower responses to influenza vaccination were reported in CMV-seropositive compared to CMV-seronegative adults and elderly, beneficial effects of CMV infection were observed as well. The lack of consensus in literature on the effect of latent CMV infection on influenza vaccination may be due to the presence of pre-existing immunity to influenza in these studies influencing the subsequent influenza vaccine response. We had the unique opportunity to evaluate the effect of age and latent CMV infection on the antibody response to the novel influenza H1N1pdm vaccine strain during the pandemic of 2009, thereby reducing the effect of pre-existing immunity on the vaccine-induced antibody response. This analysis was performed in a large study population (*n* = 263) in adults (18–52 years old). As a control, memory responses to the seasonal vaccination, including the same H1N1pdm and an H3N2 strain, were investigated in the subsequent season 2010–2011. With higher age, we found decreased antibody responses to the pandemic vaccination even within this age range, indicating signs of immunosenescence to this novel antigen in the study population. Using a generalized estimation equation regression model, adjusted for age, sex, and previous influenza vaccinations, we observed that CMV infection in contrast did not influence the influenza virus-specific antibody titer after H1N1pdm vaccination. Yet, we found higher residual protection rates (antibody level ≥40 hemagglutinin units (HAU)) in CMV-seropositive individuals than in CMV-seronegative individuals 6 months and 1 year after pandemic vaccination. In the subsequent season, no effect of age or CMV infection on seasonal influenza vaccine response was observed. In conclusion, we observed no evidence for CMV-induced impairment of antibody responses to a novel influenza strain vaccine in adults. If anything, our data suggest that there might be a beneficial effect of latent CMV infection on the protection rate after novel influenza vaccination.

## Introduction

Aging of the population poses an important public health problem. With age, the function of the human immune system declines, a phenomenon also referred to as immunosenescence (1). Profound changes of the immune system include the gradual loss of naïve cells, increase of memory cell numbers, and decreased diversity of the T cell and B cell repertoire (1–3). These changes contribute to reduced protection against infectious diseases and reduced vaccine responses in older adults. Indeed, the incidence of influenza virus infections is increased and accompanied with more complications and higher mortality in older adults (4, 5). Most developed countries recommend yearly influenza vaccination in individuals above 60 or 65 years of age (6), in order to prevent influenza virus infection by the induction of protective antibodies (4, 7). However, the antibody response to influenza vaccination in older adults is impaired, causing a suboptimal protection in this vulnerable group (7–9).

Accumulating evidence indicates that latent cytomegalovirus (CMV) infection is associated with age-related changes of the immune system, and might enhance immunosenescence (2, 10, 11). CMV is a common β-herpesvirus with a prevalence of 45–100% worldwide, which increases with advancing age (12). CMV infection causes morbidity and mortality in severely immunocompromised patients, while the virus rarely causes clinical symptoms in healthy individuals. Despite the ability of the immune system to control primary infection, the virus establishes a latent infection, with episodes of viral reactivation during lifetime (13). The frequent reactivation of CMV causes continuous antigenic stress for the immune system (3). Anti-CMV IgG levels increase with age (14–16) and are thought to increase after viral reactivation episodes, thereby reflecting the amount of experienced CMV antigenic stress during lifetime (12, 14, 17). The profound effect of CMV infection on the immune system is especially shown by the progressive large expansion of oligoclonal CMV-specific CD8 T cells and, to a lesser extent, CD4 T cells. Furthermore, CMV-seropositivity is strongly associated with an inverted CD4/8 ratio (18), bias of the TCR repertoire (19), and an increase of highly differentiated T cells (20).

It has been suggested that CMV-enhanced immunosenescence could impair the immune response to influenza vaccination (21, 22). Indeed, in several studies, CMV-seropositivity or a high anti-CMV IgG titer was associated with lower antibody responses to influenza vaccination in both adults (23–25) and older adults (25–28). However, others did not find an effect of CMV infection (29, 30), or reported even an enhanced antibody response to influenza vaccination in both young (31, 32) and older CMV-seropositive individuals (33).

The overall impact of latent CMV infection on the antibody induction by influenza vaccines remains controversial and depends, among other factors, on pre-existing immunity to influenza virus (34). Most studies investigated the antibody response to seasonal influenza vaccination; a yearly recommended trivalent influenza vaccine that often contains overlapping influenza vaccine strains in consecutive years. Natural exposure to influenza virus and previous vaccination causes pre-existing immunity, which influences the consecutive vaccine response. Higher pre-vaccination antibody titers (pre-titers) indeed were shown to result in lower post-vaccination antibody titers to subsequent vaccination (7, 35). Furthermore, one could expect a larger effect of immunosenescence on *de novo* immune responses (36, 37). A seasonal influenza vaccination is, therefore, a suboptimal study setting to investigate the effect of latent CMV infection on influenza vaccine antibody response.

We hypothesize that the effect of latent CMV infection on the antibody response to influenza vaccination can best be studied when a novel influenza virus strain is introduced into a naïve population. In this study, we had the unique opportunity to investigate the effect of latent CMV infection on the antibody response during the pandemic season of 2009 to the novel H1N1pdm vaccine strain in a large study population and at multiple time points after vaccination. This allowed a sophisticated study design to test the effect of latent CMV infection on a *de novo* influenza vaccine response by minimizing pre-existing immunity due to previous exposure by vaccination or natural infection. As a control, the influence of latent CMV infection on the memory antibody response to the vaccination in the subsequent year was also investigated, which included both the same H1N1pdm vaccine strain and an H3N2 vaccine strain.

## Materials and Methods

### Study Population and Design

The current study is embedded in a trial that evaluated the immune responses to pandemic and seasonal influenza vaccination that was conducted in 2009–2011 (the Pandemic influenza vaccination trial, Netherlands Trial Register NTR2070). This study was carried out in accordance with the recommendations of Good Clinical Practice with written informed consent from all subjects. All subjects gave written informed consent in accordance with the Declaration of Helsinki. The protocol was approved by the Central Committee on Research Involving Human Subjects of the Netherlands. Healthy individuals, between 18 and 52 years of age, were recruited among health care workers in the Utrecht area in the Netherlands. Individuals over 52 years of age were not included because of potential pre-existing immunity due to exposure to the influenza A/H1N1 strain that circulated until 1957 (38). Serum samples and questionnaires were used from the vaccine group of the Pandemic influenza vaccination cohort.

### Vaccines

**Figure 1:**
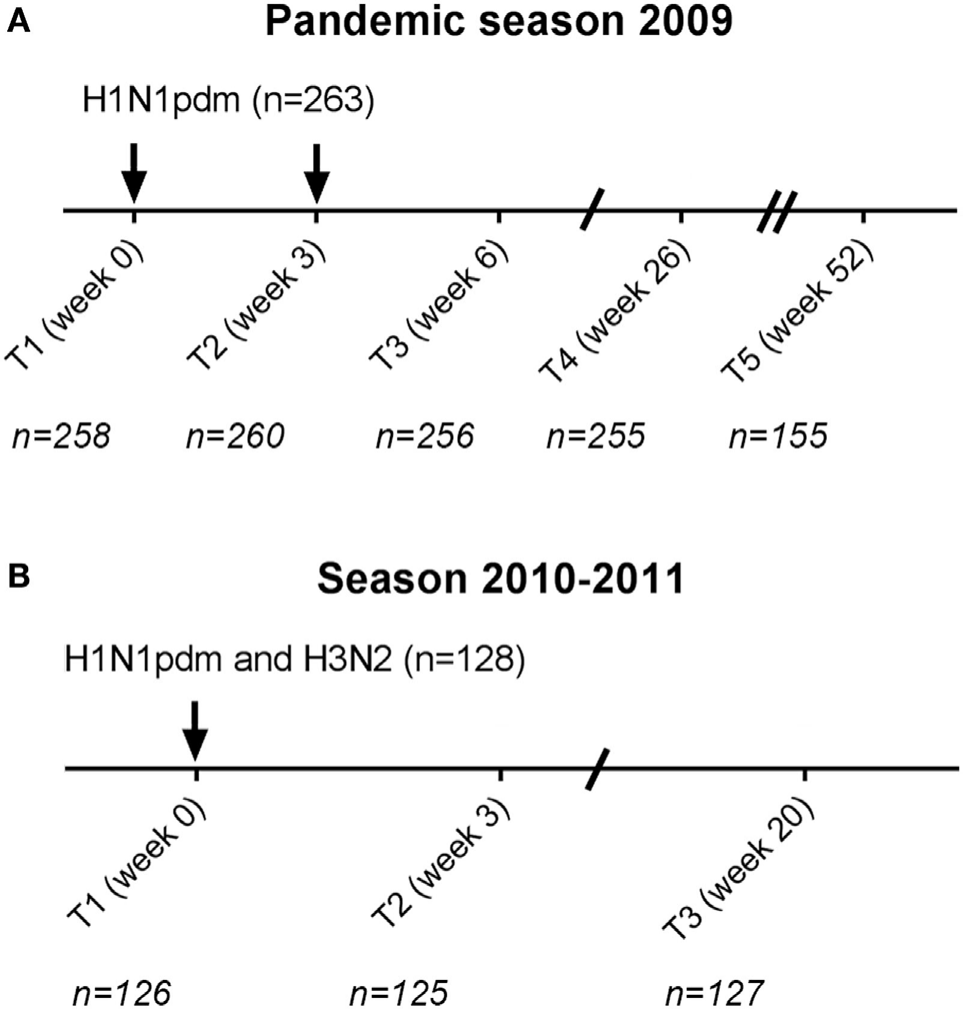
Study schedule. Participants received in the pandemic season two monovalent influenza H1N1pdm vaccinations with a 3-week interval **(A)**. In total, 263 cytomegalovirus (CMV)-seropositive and CMV-seronegative individuals were vaccinated. 155 participants continued for the subsequent year in the study (T5 season 1). In the season 2010–2011, 128 individuals were vaccinated (T1 season 2) with the seasonal trivalent influenza vaccination which contained among others the same H1N1pdm vaccine strain and an H3N2 vaccine strain **(B)**. Arrows (↓) indicate the moment of vaccination. Time points (T) indicate the moment of blood withdrawal. For each time point, the number (*N*) of individuals with data of influenza antibody levels is indicated.

In the pandemic season, individuals received two doses of the monovalent MF59-adjuvanted influenza vaccine containing influenza A/California/7/2009(H1N1pdm09) with a 3-week interval (Focetria, Novartis, Italy). Blood samples were collected before vaccination (T1), 3 weeks after vaccination at which also the second pandemic vaccine dose was given (T2), 6 weeks after the first vaccination (T3), 26 weeks after the first vaccination (T4), and if participants continued with the study during the 2010–2011 season, also 52 weeks after the first vaccination (T5) (Figure 1). Self-reported vaccine history (2006–2009) was extracted from the questionnaires. If study subjects received seasonal trivalent vaccination in 2009–2010 (Solvay, the Netherlands), it took place at least 3 weeks prior to the study or at the end of visit at time point 3 of the study. In season 2010–2011, individuals received the seasonal trivalent subunit vaccine Influvac 2010–2011, containing the influenza A vaccine strains A/California/7/2009(H1N1pdm09) and A/Perth/16/2009(H3N2) (Solvay, the Netherlands). Blood was collected before vaccination (T1), 3 weeks after vaccination (T2), and 20 weeks after vaccination (T3).

### Assessment of Serum Anti-CMV Antibody Titers

Anti-CMV IgG antibody concentrations were measured using a commercial ELISA (IBL international GMBH, Hamburg, Germany) according to manufacturer’s instructions. Participants with a CMV antibody level of ≥12 U/ml or higher were considered CMV-seropositive, a level of ≤8 U/ml were considered CMV-seronegative, and a level between 8 and 12 U/ml was considered equivocal and these participants were excluded for further analysis. CMV-seropositive individuals were divided into low anti-CMV levels (≤30 U/ml), medium anti-CMV levels (>30 U/ml, ≤90 U/ml), or high anti-CMV levels (>90 U/ml) according to the standards in the CMV ELISA kit.

### Hemagglutination-Inhibition (HI) Assay

Hemagglutination-inhibition assays were performed in the pandemic season for A/California/7/2009(H1N1pdm09) and in season 2010–2011 for A/California/7/2009(H1N1pdm09) and A/Perth/16/2009(H3N2) to determine influenza virus-specific antibody titers before and after vaccination. Briefly, a dilution series of cholera filtrate-treated serum samples was incubated with four hemagglutinin units (HAU) of influenza virus for 20 min, 0.25% turkey erythrocytes for 45 min and scored for agglutination (39). The influenza antibody titer is the inverse of the last dilution of the serum that completely inhibited hemagglutination. A detectable influenza antibody body titer is defined as >5 HAU.

### Statistical Analysis

Antibody responses to H1N1pdm influenza vaccination in the pandemic season were expressed in two different ways: (1) influenza antibody titer and (2) protection rate (antibody titer ≥40 HAU). For all statistical analyses, influenza antibody titers were log (base 2) transformed, and presented as geometric mean titer (GMT) with 95% confidence interval (CI) in the figures.

First, a two-tailed Student’s *t*-test (for two groups) or one-way ANOVA (for three or more groups) was used to explore group differences in influenza antibody titers (e.g., between low, medium, and high CMV IgG groups). For the two-tailed *T*-test, equality of variances was tested with Levene’s test for equality. Group differences in categorical variables were compared with the Fisher exact test.

Second, we investigated the effect of latent CMV infection in a multivariate context; the effect of CMV infection on influenza antibody titers was adjusted for potential confounders using a generalized estimation equation (GEE) regression model (Table S1 in Supplementary Material) (40). This model takes repeated measurements for the same individuals into account. For the continues variable outcome (influenza antibody titer) the normal distribution and for the categorical variable (influenza protection rate) the binomial distribution of the model was used. The effect of CMV infection was investigated in two ways: (a) CMV-serostatus: CMV-seropositive individuals were compared to CMV-seronegative individuals and (b) anti-CMV IgG groups: low, medium, and high anti-CMV IgG levels were compared within CMV-seropositive individuals. Confounders included were age, sex, time, and various variables concerning vaccination history (see Table S1 in Supplementary Material). The model yielded a beta regression coefficient for each variable, which reflects how a category (e.g., highest age group) compares to the reference category (e.g., lowest age group). Regression coefficients of the GEE models are given in Tables S2–S7 in Supplementary Material. The model also yielded adjusted results (i.e., influenza antibody titers or protection rates) for each time point at which comparisons between CMV-serostatus or anti-CMV IgG group were performed, by including an interaction term between time and CMV-serostatus or anti-CMV IgG group in the GEE models. The adjusted outcomes of the models and pairwise comparisons are presented in the figures. These analyses were also performed for the influenza vaccine response in season 2010–2011 for H1N1pdm and H3N2. *P* values of ≤0.10 were considered a trend and of ≤0.05 were considered significant. Data were analyzed using SPSS statistics 22 for Windows (SPSS Inc., Chicago, IL, USA) and R 3.4.0 (https://www.r-project.org/).

## Results

### Characteristics of the Study Population

In total, 288 individuals were vaccinated with the pandemic influenza vaccine in the pandemic season (Figure 1). CMV-serostatus was determined and 25 individuals with an equivocal CMV status were excluded from further analysis. Of the remaining 263 individuals, 171 were CMV-seropositive (65%). Groups of CMV-seropositive and CMV-seronegative individuals were comparable for sex, age, and previous influenza vaccinations (Table 1). In season 2010–2011, 128 of the 263 participants were vaccinated with the seasonal vaccination of which 76 (59.4%) were CMV-seropositive. Also, in the subsequent season, no differences in sex, age, and previous influenza vaccinations between CMV-seropositive and CMV-seronegative individuals were observed (Table 1).

**Table 1:** Characteristics of study population for pandemic season and season 2010–2011.

	Pandemic season				Season 2010–2011			
	Total (*n* = 263)^a^	CMV+ (*n* = 171)	CMV− (*n* = 92)	Significance	Total (*n* = 128)	CMV+ (*n* = 76)	CMV− (*n* = 52)	Significance
Age (mean and SD)	39.9 (7.8)	39.3 (8.6)	39.5 (8.5)	*P* = 0.88	41.3 (8.1)	41.7 (7.7)	40.63 (8.6)	*P* = 0.43
Sex (% men)	45.2%	51.1%	42.1%	*P* = 0.19	48.4%	46.1%	51.9%	*P* = 0.59
Previous influenza vaccination before pandemic season	49.0%	49.7%	47.8%	*P* = 0.80	65.6%	63.2%	69.2%	*P* = 0.57
Seasonal vaccination 2009–2010 before study	23.6%	23.4%	23.9%	*P* = 1.00	87.1%^b^	77.6%	78.8%	*P* = 1.00
Seasonal vaccination 2009–2010 during study	37.3%	38.0%	35.9%	*P* = 0.79				

*Cytomegalovirus (CMV)-seropositive and CMV-seronegative group are compared with Student’s *t*-test for age and with the Fischer exact test for categorical variables*.

*^a^Time point 5, 52 weeks after pandemic influenza vaccination, blood was collected of 155 participants who continued in the study for season 2010–2011*.

*^b^Seasonal vaccination in 2009 before study or during study combined*.

### Negative Effect of Age on Influenza Titers after *De Novo* Pandemic Influenza Vaccination

**Figure 2:**
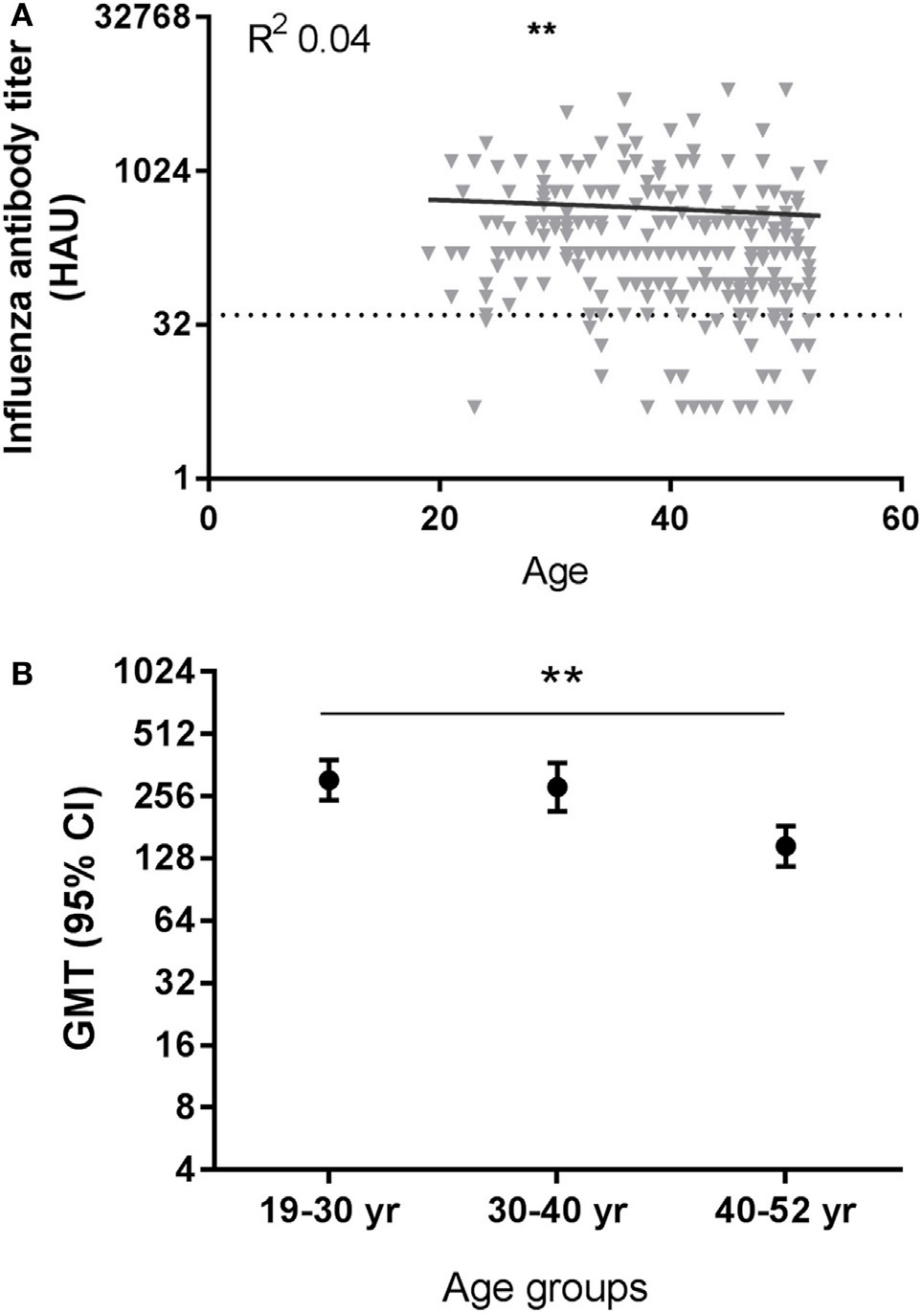
Effect of age on influenza virus-specific antibody titers after influenza vaccination. A representative figure of influenza H1N1pdm antibody titers after pandemic vaccination plotted against age (T2) **(A)**. Dotted horizontal line represents a protective influenza titer of 40 hemagglutinin units (HAU). The geometric mean of the influenza antibody titer **(B)** after vaccination is given for different age groups after vaccination with H1N1pdm for T2 (*p* = 0.016 ANOVA). Correlations are tested with Pearson correlation. Differences between two age groups are tested with Student’s *t*-test for log-transformed influenza antibody titers. **p < 0.010. GMT, geometric mean titers.

We investigated if there was an effect of age on the induction of antibodies to the pandemic influenza vaccination in our study population. After pandemic vaccination, H1N1pdm influenza virus-specific antibody titers were negatively correlated with age at all time points post-vaccination except T5 (see Table S8 in Supplementary Material). Representative data are depicted for T2 in Figure 2A (T2, *p* = 0.0013, *R* = −0.198). Individuals are divided into three age groups for further analysis by approximately 10-year intervals. Significant differences were also observed between age groups in the H1N1pdm titers (e.g., T2, *p* = 0.016), with lower responses in the oldest age group compared to the youngest two age groups (e.g., T2, 19–30 versus 40–52 year *p* = 0.007) (Figure 2B) (see Table S8 in Supplementary Material). Similar results were observed for the different age groups when analyzing the data by protection level, defined by reaching a titer of ≥40 HAU (data not shown). These data indicate that there are already signs of immunosenescence-driven impaired vaccine responses to a novel antigen challenge in middle-aged individuals.

### No Effect of CMV-Seropositivity on Antibody Titers after Pandemic Influenza Vaccination

**Figure 3:**
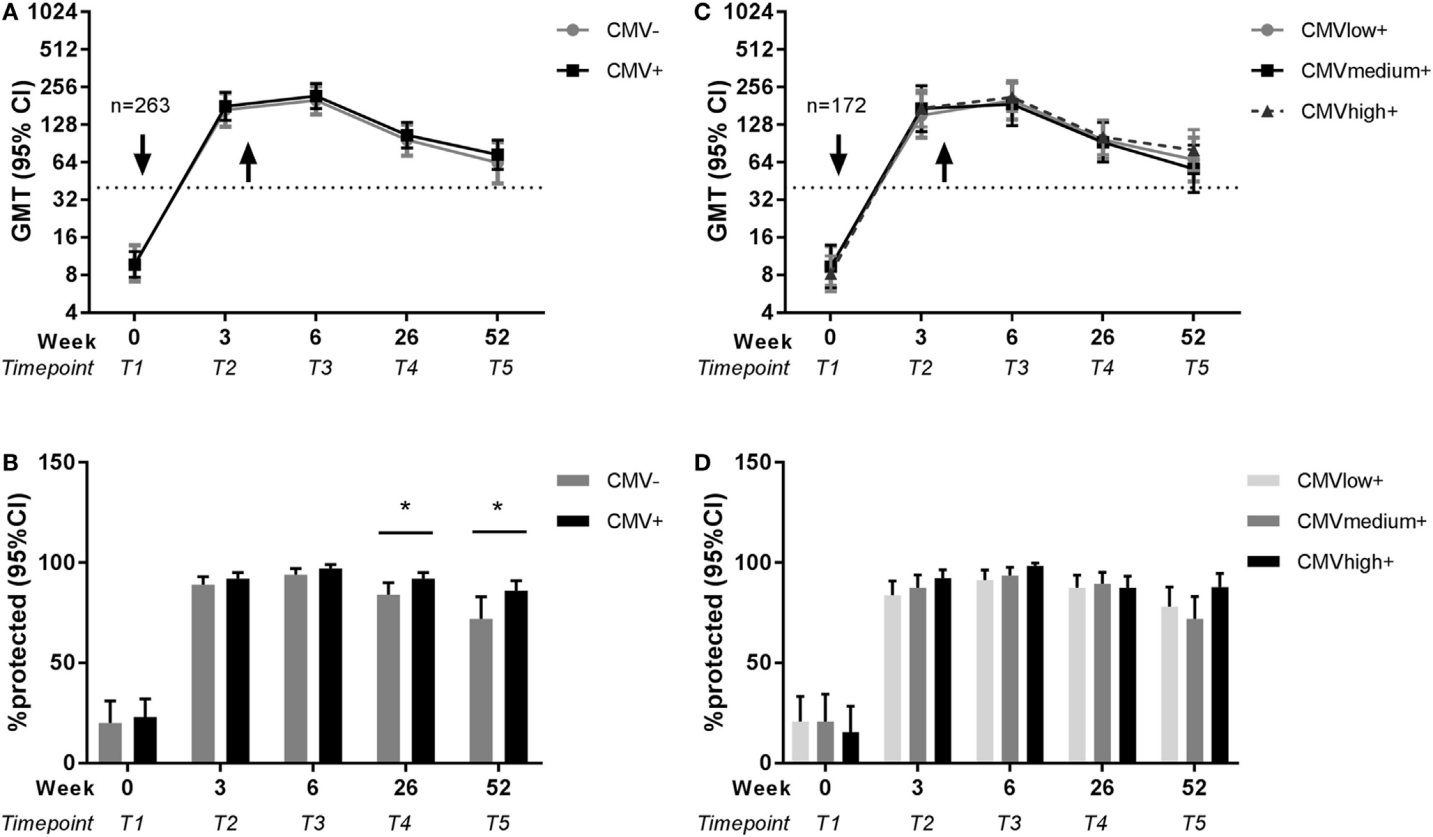
Effect of latent cytomegalovirus (CMV) infection on influenza virus-specific antibody titer and protection rate to pandemic influenza infection. Geometric mean and 95% confidence interval (CI) of influenza antibody titers and the percentage protected (defined as a titer ≥40 HAU) are shown for CMV-seropositive and CMV-seronegative individuals **(A,B)** and for CMV-seropositive individuals with low, medium, and high anti-CMV IgG levels **(C,D)** before and after pandemic vaccination with H1N1pdm in 2009. Arrows (↓) indicate the moment of vaccination. Dotted horizontal line represents a protective influenza titer of 40. Results are adjusted for sex, age group, and previous influenza vaccinations by a generalized estimation equation (GEE) regression model. Significant differences are tested by pairwise comparison between CMV-seropositive and CMV-seronegative individuals or anti-CMV IgG group high and low per separate time point. *p < 0.05.

Next, the effect of latent CMV infection on the influenza virus-specific antibody response to the vaccine with the newly introduced H1N1pdm influenza vaccine strain was investigated. CMV-seropositive individuals were compared to CMV-seronegative individuals for influenza titers before and after vaccination. No differences between CMV-seropositive and CMV-seronegative individuals in influenza titer at any time point in both seasons were found (Figure S1A in Supplementary Material). Some individuals did already show a detectable pandemic titer before vaccination, although on average the pre-titer was very low (GMT 9.4 HAU). To correct for this and other potential confounders, influenza titers of CMV-seropositive and CMV-seronegative individuals were analyzed adjusted for pre-titer, sex, age, and previous influenza vaccinations with a GEE model (Table S2 in Supplementary Material). No significant differences were found between CMV-seropositive and CMV-seronegative individuals in antibody titers at each individual time point (Figure 3A). So although age shows a negative effect on the novel pandemic H1N1pdm antibody response indicative of immunosenescence to *de novo* response (Figure 2), no effect of CMV-serostatus on the influenza virus titer is observed after pandemic vaccination in adults (Figure 3A).

### Higher Residual Protection Rates after Pandemic Influenza Vaccination in CMV-Seropositive Individuals than in CMV-Seronegative Individuals

Subsequently, we investigated whether there was an effect of CMV-serostatus on the protection rate, as defined by antibody titer ≥40 HAU, against influenza virus after influenza vaccination (41). Shortly after vaccination, no effect of CMV-serostatus on the protection rate was observed. However, CMV-seropositivity was associated with enhanced 6 months and 1 year protection rates after pandemic vaccination. The percentage influenza protected individuals is significantly higher for CMV-seropositive individuals than for CMV-seronegative individuals, both 26 weeks (*p* = 0.047) and 52 weeks (*p* = 0.044) after pandemic vaccination (Figure 3B) (unadjusted data in Figure S1B in Supplementary Material). Together, this suggests that latent CMV infection did not impair the protection rate after influenza vaccination, but if anything, might be beneficial for persistence of protection after the *de novo* influenza vaccination.

### High Anti-CMV IgG Levels As Surrogate Marker of CMV Reactivation Are Not Associated with Impaired Pandemic Influenza Vaccine Response in CMV-Seropositive Individuals

To study in the CMV-seropositive individuals whether the frequency of CMV reactivation has a negative effect on the influenza antibody responses, anti-CMV IgG levels were used as a surrogate marker of CMV reactivation (25, 42) and associated with the influenza antibody response to vaccination. To this end, CMV-seropositive individuals with low anti-CMV IgG levels (≤30 U/ml), medium anti-CMV IgG levels (>30 U/ml, ≤90U/ml) or high anti-CMV IgG levels (>90 U/ml) were compared for their influenza antibody titer and protection rate both unadjusted (Figures S1C,D in Supplementary Material) and with the GEE model (Table S3 in Supplementary Material). No differences were observed between anti-CMV IgG groups in the H1N1pdm influenza titers or protection rate after the pandemic vaccination (Figures 3C,D). This indicates that despite a negative effect of age on the antibody response to the pandemic vaccination (Figure 2), no signs of impairment by CMV reactivation were observed. Also this shows that the positive effect of CMV status on long-term protection after pandemic influenza vaccination (Figure 3B) could not be explained by differences in anti-CMV IgG groups within CMV-seropositive individuals.

### No Effect of Age or CMV-Serostatus on Seasonal Influenza Vaccination with H1N1pdm and H3N2

**Figure 4:**
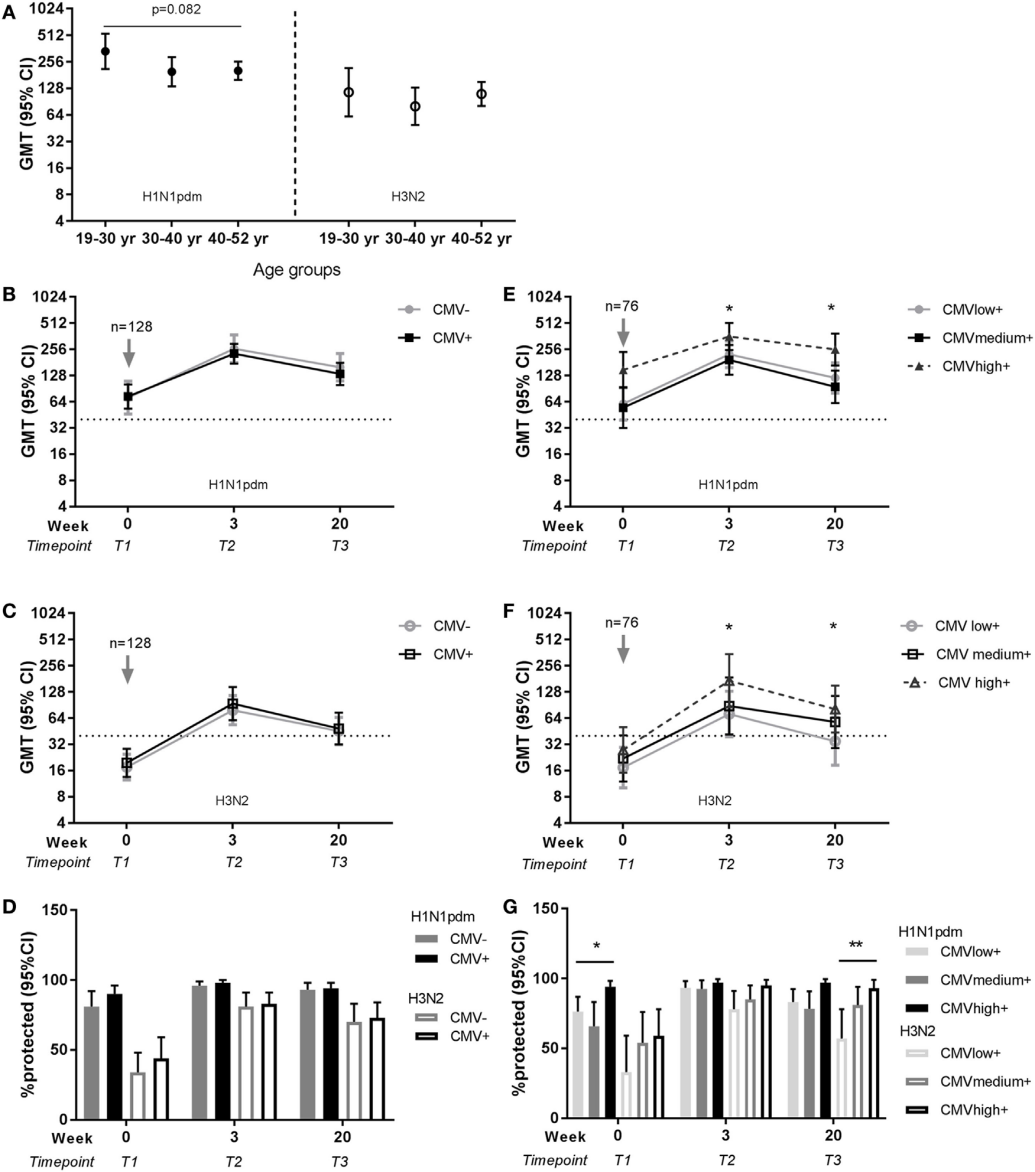
Effect of age and latent cytomegalovirus (CMV) infection on influenza virus-specific antibody titer and protection rate to seasonal influenza infection. Geometric mean and 95% confidence interval (CI) of influenza antibody titers are shown per age group for H1N1pdm (left panel) and H3N2 (right panel) for the representative time point T2 (3 weeks) after seasonal influenza vaccination 2010–2011 **(A)**. Geometric mean and 95% CI of influenza antibody titers **(B,C)** and the percentage protected [defined as a titer ≥40 hemagglutinin units (HAU)] **(D)** are shown for CMV-seropositive and CMV-seronegative individuals for H1N1pdm and H3N2 strain before and after seasonal vaccination 2010–2011. For CMV-seropositive individuals with low, medium, and high anti-CMV IgG levels geometric mean and 95% CI of influenza antibody titers **(E,F)** and the percentage protected (defined as a titer ≥40 HAU) **(G)** are shown for H1N1pdm and H3N2 strain before and after seasonal vaccination 2010–2011. Arrows (↓) indicate the moment of vaccination. Dotted horizontal line represents a protective influenza titer of 40 HAU. Results for the effect of latent CMV infection are adjusted for sex, age group, and previous influenza vaccinations by a generalized estimation equation (GEE) regression model. Significant differences are tested by pairwise comparison between CMV-seropositive and CMV-seronegative individuals or anti-CMV IgG group high and low per separate time point. Significant differences between age groups were tested with ANOVA and differences between two age groups are tested with Student’s *t*-test for (log transformed) antibody titers. *p < 0.05.

The same analyses for the effect of age and latent CMV infection on influenza vaccination were performed for the 128 individuals that continued with the study and were vaccinated in season 2010–2011 with the seasonal influenza vaccination containing the same H1N1pdm strain and an H3N2 strain. A trend of a negative effect of age on the H1N1pdm memory response was observed, but no significant differences in antibody titers for H1N1pdm or H3N2 were found between age groups at any time point after vaccination in season 2010–2011 (e.g., T2, respectively, *p* = 0.101 and *p* = 0.434) (Figure 4A). Both the influenza antibody titer and the protection rate did not differ between CMV-seropositive and CMV-seronegative individuals (Figures 4B–D; Tables S4 and S6 in Supplementary Material). Surprisingly, influenza antibody titers and protection rate after the seasonal vaccination were higher for both H1N1pdm and H3N2 in the high anti-CMV IgG levels group compared to low anti-CMV IgG levels group within the CMV-seropositive individuals at most time points after vaccination (Figures 4E–G; Tables S5 and S7 in Supplementary Material). In summary, although no clear effect of age or CMV-serostatus, high anti-CMV IgG levels seem to be associated with high influenza antibody titers and protection rate in CMV-seropositive individuals.

## Discussion

In this study, we investigated the effect of age and latent CMV infection on the antibody response to a novel influenza vaccine strain in healthy adults. We found evidence of immunosenescence in these adults from the age of 40. However, latent CMV infection did not impair the antibody responses to a *de novo* influenza vaccine response. Interestingly, indications for the contrary were observed: CMV-seropositive individuals even showed a higher long-term influenza protection rate after pandemic influenza vaccination. These results suggest that latent CMV infection does not always further weaken age-related impaired immunity, but if anything, might be beneficial.

Our study showed no negative association between latent CMV infection and the antibody response to influenza vaccination. Other studies did report negative effects in adults (23, 25, 32) and older adults (25, 26, 28, 43). However, most of these studies investigated the effect of latent CMV infection on the influenza vaccine response in the presence of pre-existing immunity. In one study, all subjects were even seroprotected (influenza antibody titer >40 HAU) before influenza vaccination (26). It is known that individuals with high pre-titers show a lower increase in influenza antibody response after influenza vaccination (7, 19, 35, 44). Therefore, high pre-titers are associated with lower seroconversion (antibody titer ≥40 HAU and ≥4-fold increase) and higher protection rate (>40 HAU). Furthermore, in all but two studies (25, 43), vaccine history was not taken into account, while previous vaccination is associated with lower seroconversion independently of pre-titers (7). Not accounting for pre-existing immunity in influenza vaccine responses, therefore, may obscure findings and lead to different findings on the effect of latent CMV infection. Here, we controlled for pre-existing immunity by investigating the effect of latent CMV infection on pandemic vaccination for which pre-existing immunity was low, and by performing analysis adjusted for pre-titers and vaccine history. By doing so, we found that influenza vaccine responses are not impaired by latent CMV infection. If anything, signs of enhanced persistence of protection after influenza vaccination were observed in CMV-seropositive individuals. We observed similar results when we analyzed the effect of latent CMV infection on the seroconversion rate. No impairment by CMV-latent infection on the vaccine response was found, but CMV-seropositive individuals showed a higher seroconversion rate 6 months and 1 year after vaccination (T4, *p* = 0.044; T5, *p* = 0.02) (data not shown).

A beneficial effect of latent CMV infection on the immune system has been indicated (10) and is suggested to reflect higher activation status of innate cells after primary CMV infection or reactivation. Accordingly, an increased antibody titer short term after influenza vaccination in young CMV-seropositive compared to young CMV-seronegative individuals was observed (31–33) and suggested to depend on boosting by low-grade inflammation and high levels of circulating IFNγ in CMV-seropositive young individuals (31, 33). A beneficial effect of latent CMV infection on the long-term persistence of protection after vaccination in adults has to our knowledge not been reported. Waning of protection is thought to be most significant in individuals above 65 years of age (45) and accelerated by latent CMV infection (46). Our results might suggest a positive effect of CMV infection in adults on the protection rate. Thereby our data fit in a scenario in which latent CMV infection has a beneficial effect in adults and may become detrimental with aging.

Two studies that reported a short-term negative effect of latent CMV infection in adults did take the factor pre-existing immunity into account by either correcting for antibody titers pre-vaccination (24) or by investigating the effect of latent CMV infection on the novel pandemic vaccine (23). However, these studies differ from our study in terms of vaccine type and analysis of the antibody response. Turner et al. studied the fold increase of influenza antibody titers to seasonal vaccination, corrected for pre-titers before vaccination (24). They reported a negative effect on the influenza antibody fold increase in one strain of the trivalent vaccine in CMV-seropositive adults with high anti-CMV IgG levels compared to CMV-seronegative adults. Wald et al. (23) also reported a negative effect of CMV-seropositivity in adults, by investigating the same pandemic H1N1pdm vaccine response in 2009 as we did. However, they did not adjust for confounders in the analysis (23). These differences in findings of the effect of latent CMV infection on the influenza vaccine response without pre-existing immunity are unexplained. We speculate that the vaccine dose and adjuvant use may be a reason for these differences. In Turner et al., half the recommended dose was used (24). Likewise, an unadjuvanted monovalent vaccine (47) was used in Wald et al., while in our study the vaccine was adjuvanted. The use of MF59 adjuvant is expected to activate the CD4+ T cells and further enhance antibody production, thereby eliciting a stronger immune response compared to an unadjuvanted vaccine. Taken together, it may be possible that only with less potent influenza vaccines, a short-term negative effect of latent CMV infection is present.

The correlation of lower antibody response to the novel pandemic influenza vaccination with age points to an immunosenescence-driven weakened immune response. Typically, lower antibody responses to influenza vaccination are associated with high age (>60 years old). Interestingly, we observed already an effect of age in this group of non-elderly (18–52 years of age), although small. This effect of age was due to a lower influenza antibody response from the age of 40 years onward. It is suggested that differences between age groups to influenza vaccination responses might also explained by HA imprinting (48). HA imprinting implicates that the immune response is skewed to the group of HA antigens of the influenza strain that is first encountered during childhood. However, this was not the case and HA imprinting could be excluded as an explanation for the age differences.

Similar analyzes were performed for the effect of age and latent CMV on the seasonal influenza vaccine response in season 2010–2011. Seasonal vaccination in 2010–2011 contained the same H1N1pdm strain of the pandemic season and the antigen-drifted H3N2 strain that overlaps in serological response to great extent with previous H3N2 strains (49). Thus, both seasonal strains elicit an immunological memory response. Immunosenescence mainly affects the *de novo* immune responses (36, 37). In line with this, effects of age on an influenza vaccine response diminish after further vaccination with the same strain (34), explaining the different findings for the effect of age between the pandemic season and season 2010–2011. It was surprising to find that individuals with high anti-CMV IgG levels showed a higher influenza titer and protection rate to seasonal vaccination. We cannot exclude that these individuals might be high-antibody producers in general, as previously shown for respiratory syncytial virus and the response to other respiratory viruses (50). Also, the total group that continued to season 2010–2011 with the study was smaller (*n* = 128) and had a higher number of previous vaccinations than the group of the pandemic season (*n* = 263), complicating the adjusted analysis. Different results were obtained for using seroconversion rate instead of protection rate as definition of responder on the seasonal vaccination. A positive effect of high anti-CMV levels group was not observed on the seroconversion rate (data not shown). This shows the importance for correcting in our statistical model for these factors and strongly implies caution with interpretations of CMV-induced effects in small study groups or non-adjusted studies as reported in literature.

Important strengths of our study compared to others are the use of a novel influenza vaccine strain, the relatively large groups of study subjects in the pandemic season and the adjusted analysis with the GEE model. Since aging and latent CMV infection are thought to affect the immune system both independently and by interacting with each other, separation of these factors in analysis is crucial (51). A limitation of the study is that the study population consists of health care workers who received repeated previous influenza vaccinations. Individuals with repeated previous seasonal influenza vaccinations show in general higher pre-vaccination titers than first-time vaccinated individuals (44). Even in the pandemic season, cross reactivity was reported for the H1N1pdm strain (52, 53). Together with potential natural exposure to the H1N1pdm strain just before the study, this may explain the detectable titers before pandemic vaccination in this study. The seasonal 2009 vaccination 3 weeks before the study in the pandemic season indeed increased the pandemic pre-titer (data not shown). However, vaccine history of the past years preceding the vaccine trial of the study subjects was reported and was adjusted for in the analysis. Importantly, pre-titers did not affect the study results, since individuals in our study without detectable pre-titers (*n* = 203) for pandemic influenza vaccination showed comparable results for the effect of CMV infection for the pandemic season (data not shown).

The influenza response in humans is complex and raises the question if influenza vaccination is the best model to investigate the effect of latent CMV infection on vaccine responses A less complicated model, in which a vaccine for people that are truly naïve is used, might be a better study design for this question. However, we consider that influenza vaccination represents the most relevant because of its high societal importance. Therefore, knowledge on the effect of CMV infection on the influenza antibody response is of great importance.

In conclusion, we used a novel influenza vaccine strain to investigate the effect of age and latent CMV infection on the *de novo* immune response to influenza. We found indeed already impaired antibody responses to vaccination in adults with increasing age, but latent CMV infection did not impair the influenza virus-specific antibody response. Thereby, we show that CMV infection does not *per se* enhance the age-related impaired immunity as assumed, but if anything might give opposite effects. A model in which CMV infection boosts the immune system during adulthood, while in older adults CMV infection enhances the aging of the immune system, might be appropriate. These results are important in the decision to invest in preventing latent CMV infection in healthy individuals through strategies such as CMV vaccination.

## Ethics Statement

This study was carried out in accordance with the recommendations of Good Clinical Practice with written informed consent from all subjects. All subjects gave written informed consent in accordance with the Declaration of Helsinki. The protocol was approved by the Central Committee on Research Involving Human Subjects of the Netherlands.

## Author Contributions

SB, DB, and JB conceptualized the study. SB, MH, and RJ executed the laboratory experiments. SB and AW performed the statistical analysis. SB, AW, DB, and JB interpreted the data and wrote the manuscript. All authors critically revised the manuscript.

## Conflict of Interest Statement

The authors declare that the research was conducted in the absence of any commercial or financial relationships that could be construed as a potential conflict of interest.
