# Supplementary material for: Negative Effect of Age, but Not of Latent Cytomegalovirus Infection on the Antibody Response to a Novel Influenza Vaccine Strain in Healthy Adults
Source: Front Immunol. 2018 Jan 29;9:82. doi: 10.3389/fimmu.2018.00082 (PMC5796903; doi:10.3389/fimmu.2018.00082)
Supplement: Supplementary file 2 [file table_1.PDF]

Dependent variable

Outcome GMT	2log titer
Outcome protection	titer ≥ 40 HAU or not
Outcome seroconversion	titer ≥ 40 HAU and 4-fold increase or not

Factor CMV effect

Factor CMV-serostatus	CMV-serostatus<br>*positive / negative*
Factor CMV IgG group in CMV + individuals	Anti-CMV IgG group<br>*Low (≤ 30U/ml) / medium (> 30 U/ml, ≤ 90 U/ml) / high (> 90 U/ml)*

Other factors

	**Season 1 (H1N1pdm)**	**Season 2 (H1N1pdm and H3N2)**
Sex	Sex<br>*Male / Female*	Sex<br>Male / Female
Age	Age group<br>*18-30 season / 30-40 season / 40-52 season*	Age group<br>18-30 season / 30-40 season / 40-52 season
Previous vaccinations	Previous influenza vaccinations in last 3 seasons<br>*Never / Sometimes / Always*	Previous influenza vaccinations in last 3 seasons<br>Never / Sometimes / Always
	Seasonal influenza vaccination 2009 before study<br>*Yes / No*	Seasonal vaccination 2009<br>Yes / No
	Seasonal influenza vaccination 2009 during study<br>*Yes / No*	

SUPPLEMENTARY TABLE 1 | Factors in Generalized Estimation Equation regression model for adjusted analysis of the effect of latent CMV infection on the antibody response to influenza vaccination. GEE models to correct for sex, age and previous influenza vaccinations are performed with one CMV factor in it (either CMV-serostatus or anti-CMV IgG level group) for one dependent variable (either titer or protection), to investigate the effect of latent CMV infection on the antibody response to influenza vaccination.
