# Supplementary material for: Negative Effect of Age, but Not of Latent Cytomegalovirus Infection on the Antibody Response to a Novel Influenza Vaccine Strain in Healthy Adults
Source: Front Immunol. 2018 Jan 29;9:82. doi: 10.3389/fimmu.2018.00082 (PMC5796903; doi:10.3389/fimmu.2018.00082)
Supplement: Supplementary file 3 [file table_2.PDF]

	**Influenza antibody titer**			**Protection**		
**Parameter**	**B (beta)**	**Standard Error**	**Sig (P-value)**	**B (beta)**	**Standard Error**	**Sig (P-value)**
(Intercept)	3.783	0.3168	0	-1.199	0.3872	0.002
**Age group 2 (40-52 year)**	-0.629	0.2547	**<u>0.013</u>**	-0.59	0.3226	**0.068**
Age group 1 (30-40 year)	-0.014	0.2596	0.958	-0.023	0.3358	0.946
Age group 0 (18-30 year)	0a	.	.	0a	.	.
Sex male	0.161	0.1974	0.414	-0.074	0.247	0.765
Sex female	0a	.	.	0a	.	.
**Previous influenza vaccinations yes**	-0.583	0.3263	**0.074**	-0.607	0.4259	0.154
**Previous influenza vaccinations sometimes**	-0.459	0.2442	**0.06**	-0.381	0.346	0.27
Previous influenza vaccinations no	0[a]	.	.	0[a]	.	.
**Seasonal 2009 vaccination before study yes**	0.017	0.2758	0.952	0.803	0.3979	**<u>0.043</u>**
Seasonal 2009 vaccination before study no	0[a]	.	.	0[a]	.	.
Seasonal 2009 vaccination during study yes	-0.004	0.2612	0.989	-0.094	0.3367	0.779
Seasonal 2009 vaccination during study no	0[a]	.	.	0[a]	.	.
CMV-serostatus positive	-0.029	0.2395	0.903	0.189	0.3461	0.586
CMV-serostaus negative	0[a]	.	.	0[a]	.	.
**Timepoint 5**	2.679	0.29	**<u>< 0.001</u>**	2.375	0.3555	**<u>< 0.001</u>**
**Timepoint 4**	3.286	0.2388	**<u>< 0.001</u>**	3.071	0.3557	**<u>< 0.001</u>**
**Timepoint 3**	4.344	0.2405	**<u>< 0.001</u>**	4.148	0.415	**<u>< 0.001</u>**
**Timepoint 2**	4.089	0.2684	**<u>< 0.001</u>**	3.466	0.3828	**<u>< 0.001</u>**
Timepoint 1	0[a]	.	.	0[a]	.	.
CMV-serostatus positive * Timepoint 5	0.243	0.3385	0.473	0.63	0.4587	0.17
CMV-serostatus positive * Timepoint 4	0.155	0.2825	0.582	0.582	0.4633	0.209
CMV-serostatus positive * Timepoint 3	0.138	0.2892	0.632	0.613	0.6122	0.317
CMV-serostatus positive * Timepoint 2	0.122	0.3201	0.703	0.188	0.4869	0.7
CMV-serostatus positive * Timepoint 1	0[a]	.	.	0[a]	.	.
CMV-serostatus negative * Timepoint 5	0[a]	.	.	0[a]	.	.
CMV-serostatus negative * Timepoint 4	0[a]	.	.	0[a]	.	.
CMV-serostatus negative * Timepoint 3	0[a]	.	.	0[a]	.	.
CMV-serostatus negative * Timepoint 2	0[a]	.	.	0[a]	.	.
CMV-serostatus negative * Timepoint 1	0[a]	.	.	0[a]	.	.

SUPPLEMENTARY TABLE 2 | Regression table effect CMV-serostatus on pandemic influenza vaccine response of H1N1pdm strain in the pandemic season. Bold: p value < 0.10 Bold and underlined: p value < 0.05. [a] reference category
