# Supplementary material for: Negative Effect of Age, but Not of Latent Cytomegalovirus Infection on the Antibody Response to a Novel Influenza Vaccine Strain in Healthy Adults
Source: Front Immunol. 2018 Jan 29;9:82. doi: 10.3389/fimmu.2018.00082 (PMC5796903; doi:10.3389/fimmu.2018.00082)
Supplement: Supplementary file 4 [file table_3.PDF]

	Influenza antibody titer			Protection		
**Parameter**	**B (beta)**	**Standard Error**	**Sig (P-value)**	**B (beta)**	**Standard Error**	**Sig (P-value)**
(Intercept)	3.844	0.3311	0	-0.794	0.419	0.058
Age group 2 (40-52 year)	-0.485	0.3022	0.109	-0.527	0.432	0.222
Age group 1 (30-40 year)	0.438	0.3215	0.173	0.606	0.443	0.171
Age group 0 (18-30 year)	0[a]	.	.	0[a]	.	.
Sex male	-0.226	0.2302	0.326	-0.495	0.333	0.137
Sex female	0[a]	.	.	0[a]	.	.
Previous influenza vaccinations yes	-0.394	0.3869	0.309	-0.689	0.571	0.228
Previous influenza vaccinations sometimes	-0.392	0.2664	0.142	-0.251	0.417	0.548
Previous influenza vaccinations no	0[a]	.	.	0[a]	.	.
Seasonal 2009 vaccination before study yes	-0.37	0.2902	0.203	0.110	0.459	0.810
Seasonal 2009 vaccination before study no	0[a]	.	.	0[a]	.	.
Seasonal 2009 vaccination during study yes	-0.055	0.2745	0.841	-0.454	0.402	0.258
Seasonal 2009 vaccination during study no	0[a]	.	.	0[a]	.	.
CMV IgG group high	-0.208	0.335	0.535	-0.387	0.533	0.468
CMV IgG group medium	-0.019	0.3543	0.957	0[a]	.	.
CMV IgG group low	0[a]	.	.	0[a]	.	.
**Timepoint 5**	2.83	0.3081	**< 0.001**	2.922	0.527	**< 0.001**
**Timepoint 4**	3.357	0.2884	**< 0.001**	3.643	0.573	**< 0.001**
**Timepoint 3**	4.404	0.326	**< 0.001**	4.075	0.605	**< 0.001**
**Timepoint 2**	4.012	0.3443	**< 0.001**	3.314	0.511	**< 0.001**
Timepoint 1	0[a]	.	.	0[a]	.	.
CMV IgG group high * Timepoint 5	0.459	0.4242	0.279	1.147	0.767	0.135
CMV IgG group high * Timepoint 4	0.278	0.3884	0.475	0.390	0.734	0.596
**CMV IgG group high * Timepoint 3**	0.293	0.4027	0.467	2.238	1.278	**0.080**
**CMV IgG group high * Timepoint 2**	0.388	0.4392	0.377	1.275	0.738	**0.084**
CMV IgG group high * Timepoint 1	0[a]	.	.	0[a]	.	.
CMV IgG group medium * Timepoint 5	-0.226	0.4282	0.597	-0.364	0.723	0.614
CMV IgG group medium * Timepoint 4	-0.048	0.3668	0.895	0.219	0.752	0.771
CMV IgG group medium * Timepoint 3	-0.084	0.4223	0.843	0.362	0.866	0.676
CMV IgG group medium * Timepoint 2	0.195	0.4474	0.663	0.330	0.703	0.638
CMV IgG group medium * Timepoint 1	0[a]	.	.	0[a]		
CMV IgG group low * Timepoint 5	0[a]	.	.	0[a]		
CMV IgG group low * Timepoint 4	0[a]	.	.	0[a]		
CMV IgG group low * Timepoint 3	0[a]	.	.	0[a]		
CMV IgG group low * Timepoint 2	0[a]	.	.	0[a]	.	.
CMV IgG group low * Timepoint 1	0[a]	.	.	0[a]	.	.

SUPPLEMENTARY TABLE 3 | Regression table effect CMV IgG group on pandemic influenza vaccine response of H1N1pdm strain in the pandemic season. Bold: p value < 0.10 Bold and underlined: p value < 0.05. [a] reference category
