# Supplementary material for: Negative Effect of Age, but Not of Latent Cytomegalovirus Infection on the Antibody Response to a Novel Influenza Vaccine Strain in Healthy Adults
Source: Front Immunol. 2018 Jan 29;9:82. doi: 10.3389/fimmu.2018.00082 (PMC5796903; doi:10.3389/fimmu.2018.00082)
Supplement: Supplementary file 5 [file table_4.PDF]

	Influenza antibody titer			Protection		
**Parameter**	**B (beta)**	**Standard Error**	**Sig (P-value)**	**B (beta)**	**Standard Error**	**Sig (P-value)**
(Intercept)	7.139	0.3716	0	3.222	1.1526	0.005
Age group 2 (40-52 year)	-0.334	0.3094	0.281	-1.643	1.0299	0.111
Age group 1 (30-40 year)	-0.221	0.347	0.525	-1.385	1.0983	0.207
Age group 0 (18-30 year)	0[a]	.	.	0[a]	.	.
Sex male	-0.19	0.2621	0.468	-0.236	0.4394	0.591
Sex female	0[a]	.	.	0[a]	.	.
**Previous influenza vaccinations yes**	-0.934	0.481	**0.052**	-0.547	0.7594	0.471
Previous influenza vaccinations sometimes	-0.693	0.4209	0.1	-0.657	0.7267	0.366
Previous influenza vaccinations no	0[a]	.	.	0[a]	.	.
Seasonal 2009 vaccination yes	-0.315	0.4453	0.479	-0.467	0.8388	0.578
Seasonal 2009 vaccination no	0[a]	.	.	0[a]	.	.
CMV-serostaus positive	0.042	0.3729	0.911	0.685	0.4495	0.128
CMV-serostaus negative	0[a]	.	.	0[a]	.	.
**Timepoint 3**	1.158	0.2085	**< 0.001**	1.202	0.3643	**0.001**
**Timepoint 2**	1.864	0.2372	**< 0.001**	1.658	0.4823	**0.001**
Timepoint 1	0[a]	.	.	0[a]	.	.
CMV-serostatus positive * Timepoint 3	-0.298	0.2569	0.246	-0.526	0.4531	0.246
CMV-serostatus positive * Timepoint 2	-0.231	0.284	0.415	0.102	0.7427	0.891
CMV-serostatus positive * Timepoint 1	0[a]	.	.	0[a]	.	.
CMV-serostatus negative * Timepoint 3	0[a]	.	.	0[a]	.	.
CMV-serostatus negative * Timepoint 2	0[a]	.	.	0[a]	.	.
CMV-serostatus negative * Timepoint 1	0[a]	.	.	0[a]	.	.

SUPPLEMENTARY TABLE 4 | Regression table effect CMV-serostatus on seasonal influenza vaccine response of H1N1pdm strain in the season 2010-2011. Bold: p value < 0.10 Bold and underlined: p value < 0.05. [a] reference category
