# Supplementary material for: Negative Effect of Age, but Not of Latent Cytomegalovirus Infection on the Antibody Response to a Novel Influenza Vaccine Strain in Healthy Adults
Source: Front Immunol. 2018 Jan 29;9:82. doi: 10.3389/fimmu.2018.00082 (PMC5796903; doi:10.3389/fimmu.2018.00082)
Supplement: Supplementary file 6 [file table_5.PDF]

	Influenza antibody titer			Protection		
**Parameter**	**B (beta)**	**Standard Error**	**Sig (P-value)**	**B (beta)**	**Standard Error**	**Sig (P-value)**
(Intercept)	7.321	0.3907	0	3.920744	1.127556	0.001
**Age group 2 (40-52 year)**	-0.969	0.4197	**0.021**	-1.06577	0.777988	0.171
Age group 1 (30-40 year)	-0.663	0.4203	0.115	#		
Age group 0 (18-30 year)	0[a]	.	.	0[a]	.	.
**Sex male**	-0.599	0.2927	0.041	-1.35677	0.665921	**0.042**
Sex female	0[a]	.	.	0[a]	.	.
Previous influenza vaccinations yes	-0.55	0.4719	0.244	0.066665	0.996880	0.947
Previous influenza vaccinations sometimes	-0.489	0.4355	0.261	-0.52934	0.897337	0.555
Previous influenza vaccinations no	0[a]	.	.	0[a]	.	.
Seasonal 2009 vaccination yes	-0.418	0.3913	0.286	-1.34266	1.088206	0.217
Seasonal 2009 vaccination no	0[a]	.	.	0[a]	.	.
**CMV IgG group high**	1.295	0.4562	**0.005**	1.76129	0.897221	**0.05**
CMV IgG group medium	-0.149	0.5024	0.767	-0.61748	0.765129	0.42
CMV IgG group low	0[a]	.	.	0[a]	.	.
**Timepoint 3**	0.986	0.2243	0	0.495826	0.364392	0.174
**Timepoint 2**	1.887	0.2159	0	1.62821	0.810095	**0.044**
Timepoint 1	0[a]	.	.	0[a]	.	.
CMV IgG group high * Timepoint 3	-0.209	0.3584	0.56	0.258459	1.355159	0.849
**CMV IgG group high * Timepoint 2**	-0.615	0.3293	**0.062**	-0.87393	1.531723	0.568
CMV IgG group high * Timepoint 1	0[a]	.	.	0[a]	.	.
CMV IgG group medium * Timepoint 3	-0.191	0.3379	0.573	0.262801	0.586560	0.654
CMV IgG group medium * Timepoint 2	-0.064	0.417	0.878	0.50072	1.231240	0.684
CMV IgG group medium * Timepoint 1	0[a]	.	.	0[a]		
CMV IgG group low * Timepoint 3	0[a]	.	.	0[a]		
CMV IgG group low * Timepoint 2	0[a]	.	.	0[a]		
CMV IgG group low * Timepoint 1	0[a]	.	.	0[a]		

SUPPLEMENTARY TABLE 5 | Regression table effect CMV IgG group serostatus on seasonal influenza vaccine response of H1N1pdm strain in the season 2010-2011. Bold: p value <0.10 Bold and underlined: p value <0.05. # for age group 0 (18-30 year) all participants were protected after vaccination (outcome 1) wherefore the model did not run. This is solved merging age group 0 and 1 for this model, resulting in no separate regression coefficient for age group 1. [a] reference category
