# Supplementary material for: Negative Effect of Age, but Not of Latent Cytomegalovirus Infection on the Antibody Response to a Novel Influenza Vaccine Strain in Healthy Adults
Source: Front Immunol. 2018 Jan 29;9:82. doi: 10.3389/fimmu.2018.00082 (PMC5796903; doi:10.3389/fimmu.2018.00082)
Supplement: Supplementary file 7 [file table_6.PDF]

| | Influenza antibody titer | | | Protection | | |
|---|---|---|---|---|---|---|
| **Parameter** | **B (beta)** | **Standard Error** | **Sig (P-value)** | **B (beta)** | **Standard Error** | **Sig (P-value)** |
| (Intercept) | 3.937 | 0.4387 | 0 | -1.055 | 0.571 | 0.065 |
| Age group 2 (40-52 year) | 0.044 | 0.4029 | 0.913 | -0.351 | 0.5248 | 0.504 |
| Age group 1 (30-40 year) | -0.239 | 0.4554 | 0.6 | -0.516 | 0.5764 | 0.371 |
| Age group 0 (18-30 year) | 0[a] | . | . | 0[a] | . | . |
| Sex male | -0.023 | 0.2778 | 0.933 | 0.215 | 0.3255 | 0.508 |
| Sex female | 0[a] | . | . | 0[a] | . | . |
| Previous influenza vaccinations yes | -0.224 | 0.4314 | 0.604 | -0.069 | 0.4824 | 0.886 |
| Previous influenza vaccinations sometimes | -0.06 | 0.3824 | 0.875 | 0.422 | 0.4741 | 0.373 |
| Previous influenza vaccinations no | 0[a] | . | . | 0[a] | . | . |
| **Seasonal 2009 vaccination yes** | 0.729 | 0.4627 | 0.115 | 0.868 | 0.4818 | **0.072** |
| Seasonal 2009 vaccination no | 0[a] | . | . | 0[a] | . | . |
| CMV-serostaus positive | 0.163 | 0.3143 | 0.605 | 0.425 | 0.3732 | 0.255 |
| CMV-serostaus negative | 0[a] | . | . | 0[a] | . | . |
| **Timepoint 3** | 1.377 | 0.2333 | **< 0.001** | 1.548 | 0.3552 | **< 0.001** |
| **Timepoint 2** | 2.175 | 0.2797 | **< 0.001** | 2.144 | 0.4236 | **< 0.001** |
| Timepoint 1 | 0[a] | . | . | 0[a] | . | . |
| CMV-serostatus positive * Timepoint 3 | -0.065 | 0.3074 | 0.832 | -0.278 | 0.4502 | 0.537 |
| CMV-serostatus positive * Timepoint 2 | 0.088 | 0.3812 | 0.817 | -0.271 | 0.5373 | 0.614 |
| CMV-serostatus positive * Timepoint 1 | 0[a] | . | . | 0[a] | . | . |
| CMV-serostatus negative * Timepoint 3 | 0[a] | . | . | 0[a] | . | . |
| CMV-serostatus negative * Timepoint 2 | 0[a] | . | . | 0[a] | . | . |
| CMV-serostatus negative * Timepoint 1 | 0[a] | . | . | 0[a] | . | . |

**SUPPLEMENTARY TABLE 6 | Regression table effect CMV-serostatus on seasonal influenza vaccine response of H3N2 strain in the season 2010-2011.** Bold: p value <0.10 Bold and underlined: p value <0.05. [a] reference category
