# Supplementary material for: Negative Effect of Age, but Not of Latent Cytomegalovirus Infection on the Antibody Response to a Novel Influenza Vaccine Strain in Healthy Adults
Source: Front Immunol. 2018 Jan 29;9:82. doi: 10.3389/fimmu.2018.00082 (PMC5796903; doi:10.3389/fimmu.2018.00082)
Supplement: Supplementary file 8 [file table_7.PDF]

	Influenza antibody titer			Protection		
**Parameter**	**B (beta)**	**Standard Error**	**Sig (P-value)**	**B (beta)**	**Standard Error**	**Sig (P-value)**
(Intercept)	3.895	0.7973	0	-1.777	0.861	0.039
Age group 2 (40-52 year)	-0.347	0.7905	0.661	-0.428	0.817	0.601
Age group 1 (30-40 year)	-0.519	0.8256	0.53	-0.373	0.8499	0.66
Age group 0 (18-30 year)	0[a]	.	.	0[a]	.	.
**Sex male**	-0.093	0.3691	0.801	0.475	0.4621	0.304
Sex female	0[a]	.	.	0[a]	.	.
Previous influenza vaccinations yes	0.407	0.5556	0.464	0.81	0.6561	0.217
**Previous influenza vaccinations sometimes**	0.407	0.4929	0.409	1.406	0.6414	**<u>0.028</u>**
Previous influenza vaccinations no	0[a]	.	.	0[a]	.	.
Seasonal 2009 vaccination yes	0.566	0.6298	0.368	0.763	0.6701	0.255
Seasonal 2009 vaccination no	0[a]	.	.	0[a]	.	.
**CMV IgG group high**	0.674	0.3982	**0.09**	1.033	0.6058	**0.088**
CMV IgG group medium	0.35	0.5267	0.506	0.836	0.6501	0.199
CMV IgG group low	0[a]	.	.	0[a]	.	.
**Timepoint 3**	1.003	0.2659	**<u>< 0.001</u>**	0.989	0.3764	**<u>0.009</u>**
**Timepoint 2**	2.043	0.3249	**<u>< 0.001</u>**	1.965	0.5822	**<u>0.001</u>**
Timepoint 1	0[a]	.	.	0[a]	.	.
CMV IgG group high * Timepoint 3	0.554	0.4295	0.197	1.211	1.0022	0.227
CMV IgG group high * Timepoint 2	0.598	0.5711	0.295	0.741	1.1863	0.532
CMV IgG group high * Timepoint 1	0[a]	.	.	0[a]	.	.
CMV IgG group medium * Timepoint 3	0.387	0.5298	0.465	0.31	0.7386	0.675
CMV IgG group medium * Timepoint 2	-0.043	0.6415	0.946	-0.339	0.8217	0.68
CMV IgG group medium * Timepoint 1	0[a]	.	.	0[a]	.	.
CMV IgG group low * Timepoint 3	0[a]	.	.	0[a]	.	.
CMV IgG group low * Timepoint 2	0[a]	.	.	0[a]	.	.
CMV IgG group low * Timepoint 1	0[a]	.	.	0[a]	.	.

SUPPLEMENTARY TABLE 7 | Regression table effect CMV IgG group serostatus on seasonal influenza vaccine response of H3N2 strain in the season 2010-2011. Bold: p value < 0.10 Bold and underlined: p value < 0.05 [a] reference category
