# Supplementary material for: Negative Effect of Age, but Not of Latent Cytomegalovirus Infection on the Antibody Response to a Novel Influenza Vaccine Strain in Healthy Adults
Source: Front Immunol. 2018 Jan 29;9:82. doi: 10.3389/fimmu.2018.00082 (PMC5796903; doi:10.3389/fimmu.2018.00082)
Supplement: Supplementary file 9 [file table_8.PDF]

Time point	Correlation age and influenza titer	Differences between age groups in influenza titer
T2	p=0.0013, R -0.198	p=0.016
T3	p=0.000, R= -0.269	p=0.000
T4	p=0.006, R= -0.172	p=0.015
T5	p=0.165, R=-0.112	p=0.714

SUPPLEMENTARY TABLE 8 | Negative effect of age on titers after de novo pandemic influenza vaccination. Correlations between age and H1N1pdm influenza antibody titer is presented on all time points of the pandemic season (Pearson correlation). Differences H1N1pdm influenza antibody titers after vaccination (T2-T5) (log transformed) between age groups (18-30 year, 30-40 year, 40-52 year) are presented (ANOVA). $P < 0.05$ is considered significant.
